# Assessment of Nutritional Status and Correlation of Factors With Body Mass Index of Cancer Patients: A Cross-Sectional Study

**DOI:** 10.7759/cureus.54146

**Published:** 2024-02-13

**Authors:** K Vidhya, Sweety Gupta, Lekshmi R, Namitha RS, Yogapriya Velumani, Deepika Raina, Kusum Kumari, Amit Gupta

**Affiliations:** 1 Nuclear Medicine, All India Institute of Medical Sciences, Rishikesh, IND; 2 Radiation Oncology, All India Institute of Medical Sciences, Rishikesh, IND; 3 General Surgery, All India Institute of Medical Sciences, Rishikesh, IND; 4 College of Nursing, All India Institute of Medical Sciences, Deoghar, IND

**Keywords:** albumin, dietary, body mass index, cancer, malnutrition

## Abstract

Background: Decreased diet intake and malnourishment have profound implications on cancer patients' quality of life and survival. Malnutrition increases the risk of postoperative complications, increases hospital length stays, reduces patient's tolerance to radiation and chemotherapy treatment, and results in poor response to treatment. In the present study, we intended to assess the nutritional status of cancer patients and find the correlation of body mass index with anthropometric and blood parameters.

Material & methods: The study was prospective and cross-sectional, and 104 patients with newly diagnosed solid tumors were included. Patient demographics, symptoms, and anthropometric and blood parameters were collected. The correlation was estimated with Pearson’s correlation coefficient. A p-value of less than 0.05 was considered significant.

Results: The association between stages of the disease, dental status, type of diet, and BMI was p=0.701, 0.216, and 0.422, respectively, and was not statistically significant. The anthropometric parameters mid upper arm circumference (MUAC cm), mid arm circumference (MAC cm), and triceps skinfold thickness (TSF mm) correlated with body mass index (BMI kg/m^2^) and had statistically significant p values of 0.0001, 0.0001, and 0.033, respectively. The correlation was assessed between hemoglobin, red cell distribution width, neutrophil-to-lymphocyte ratio, and serum albumin levels with BMI, but except for albumin (p=0.05), no other blood parameter correlated.

Conclusion: Nutritional assessment is vital in recognizing patients at risk of treatment-associated complications and poor responders to treatment. In this study, BMI correlated with anthropometric parameters MUAC, MAMC, and TSF. Baseline dietary assessments of patients will help focus on the nutritional build-up of patients before starting treatment.

## Introduction

Cancer is one of the main reasons for morbidity and mortality throughout the world. Weight loss and nutritional problems are often associated with cancer. In advanced cancer stages, extreme weight loss is seen. Undernutrition is a hallmark of cancer. Approximately 40% of cancer patients present with weight loss [1]. Several studies indicate that malnutrition resulting from reductions in dietary intake occurs in 30 to 50% of cancer patients [1,2]. Decreased diet intake and malnourishment have profound implications on cancer patients' quality of life and survival [2]. Weight loss in cancer patients is associated with symptom distress (including fatigue, depression, and social withdrawal), poor quality of life, and increased treatment morbidity. Many cancer patients may not be candidates for potentially curative treatment because of poor nutritional status and performance status. Also, the effects of malnutrition increased the risk of postoperative complications, increased hospital length stay, reduced patient’s tolerance to radiation and chemotherapy treatment, and resulted in poor response to treatment [3]. Therefore, custom-made approaches to identify patients at nutritional risk are crucial to implementing nutritional support efficiently to reduce cancer morbidity. Cancer patients' nutritional status can be measured by history, physical assessment, and blood parameters [4,5]. This can help patients tolerate the oncology treatment effectively, improve their response to treatment, and reduce complications. In the present study, we intended to assess factors leading to decreased dietary intake and nutritional assessment of cancer patients so that we can identify patients at risk of malnourishment, help patients increase or maintain weight, and find the correlation of body mass index with anthropometric and blood parameters.

## Materials and methods

Study design

The current study design was a prospective, cross-sectional study. One hundred four patients with newly diagnosed solid tumors who were visiting Radiation Oncology and Surgery OPD between January 2021 and December 2021 were included in the present study.

The inclusion criteria for patients older than 18 years and recently diagnosed cancer patients with solid tumors. Previously treated cancer patients, disease-free patients on follow-up, and patients suffering from hematological malignancies were excluded.

Data collection procedure

After taking written informed consent, a complete history and physical examination with symptoms of all patients were recorded. Each site was staged according to the 8th AJCC (American Joint Committee on Cancer) classification or FIGO (The International Federation of Gynecology and Obstetrics) in gynecological malignancies. Section I included patient demographics questions, i.e., age, gender, comorbidities, performance status, Cancer site, dietary habits, weight loss, and ongoing medication recorded. Section II included symptom assessment by assessing the history of risk factors and symptoms affecting dietary intake. Dietary history included evaluation of symptoms such as pain, nausea, vomiting, early satiety, constipation, taste alterations, dental and oral problems, and dysphagia leading to decreased appetite. Weight loss was deﬁned as losing at least 5% of initial body weight and maintaining the loss for at least six months. Section III included anthropometric measurement for nutritional assessment. The instrument used was measuring tape and calipers. Height and weight were measured to calculate body mass index (BMI kg/m^2^), mid-upper arm circumference (cm)/ mid-arm circumference (cm), and triceps skinfold thickness (mm). Baseline blood investigations of hemoglobin, red cell distribution width (RDW), total leucocyte count, serum albumin, total protein, and serum creatinine of all patients were recorded. All the anthropometric and blood parameters and their normal values in both males and females, which were included in the study, are mentioned in Table 1.

**Table 1:** Anthropometric parameters and blood parameters' normal values

Variable	Normal Values
Body mass index (BMI)	Underweight <18.5
	Normal 18.5-25
	Overweight 25-29.9
	Obese > 30
Mid-upper arm circumference (MUAC) (cm)	Normal: >29 cm (Male)
	Normal: >28.5 cm (Female)
Mid-arm muscle circumference (MAMC) (cm)	Normal: >25 cm (Male)
	Normal: >23cm (Female)
Triceps skinfold thickness (TSF) (mm)	Normal: >12.5 mm (Male)
	Normal: >16.5 mm (Female)
Hemoglobin (gm/dl)	13.5 - 17.5 g/dL (Male)
	12.0 -15.5 g/dL(Female)
Red cell distribution width	11.5 - 14.5%
Total leucocyte count (mm^3 ^)	4-11 x 10^3 ^/mm^3^
Absolute neutrophil count	1,500 - 8,000 / mm^3^
Absolute lymphocyte count	1000 - 4000 /mm^3^
Serum albumin (gm/dl)	3.5 - 5.0 gm/dl
Total protein (gm/dl)	6.0 - 8.3 g/dL

Before patient recruitment, ethical committee approval was obtained from All India Institute of Medical Sciences Rishikesh Institute Ethical Committee (AIIMS/IEC/21/492 dated 02/09/2021). Written informed consent was obtained from all patients prior to commencement of the study.

Statistical analysis

IBM SPSS Statistics for Windows, Version 25 (Released 2017; IBM Corp., Armonk, New York, United States) was used for data analysis. Categorical variables were expressed using descriptive statistics (frequency and percentages), and continuous variables using mean and standard deviation. Pearson’s correlation coefficient was used to identify correlation. A p-value of less than 0.05 was considered significant.

## Results

Sociodemographic profile and clinical characteristics

We analyzed 104 consecutive patients with solid malignancies during the study period. The age of patients ranged from 18-84 years, with a mean of 52.7 years. The male-to-female ratio was 1.4. Most of the patients, 75(72.1%), had ECOG-1 (Eastern Cooperative Oncology Group), and only one was ECOG-4. Thirty-seven (35.6%) patients were habituated to smoking. Only seven (6.7%) patients were edentulous. The most common site of cancer was the head and neck region 41(39.4%), followed by breast cancer in 25 (24&) patients. Eighty-four (80.7%) patients had stage III and IV, i.e., advanced stages of malignancies. At the presentation time, 32(30.8%) patients were underweight and had a BMI of less than 18.5 (Table 2).

**Table 2:** Sociodemographic profile and clinical characteristics of study patients (N=104)

Variables	Options	Frequency (%)
Age (in years)	18-30	06 (5.8)
	31-40	11 (10.6)
	41-50	23 (22.1)
	51-60	31 (29.8)
	61-70	26 (25.0)
	> 70	07 (6.7)
Gender	Male	61 (58.7)
	Female	43 (41.3)
Marital status	Married	101 (97.1)
	Single	03 (2.9)
ECOG*	0	15 (14.4)
	1	75 (72.1)
	2	11 (10.6)
	3	02 (1.9)
	4	01 (1.0)
Personal Habits	Smoking	37 (35.6)
	Tobacco	07 (6.7)
	Smoking+ Alcohol+ Tobacco	16 (15.3)
	Nil	44 (42.3)
Comorbidities	Cardiac disease	03 (2.9)
	Hypertension	04 (3.8)
	Diabetes	05 (4.8)
	Others	06 (5.7)
	None	86 (82.7)
Dental status	Edentulous	07 (6.7)
	Intact	77 (74.0)
	Missing	20 (19.3)
Cancer Site	Head and Neck	41 (39.4)
	Breast	25 (24.0)
	Lung	05 (4.8)
	Gastrointestinal	15 (14.4)
	Genitourinary	06 (5.8)
	Gynaecological	06 (5.8)
	Others(CNS**/thyroid)	04 (3.8)
Stage	I	01 (01.0)
	II	18 (17.3)
	III	62 (59.5)
	IV	22 (21.2)
	Unknown	01 (01.0)
Diet	Liquid	07 (06.7)
	Semisolid	19 (18.3)
	Solid	75 (72.1)
	Tube feed	03 (02.9)
Mouth Opening	More than 35 mm	100 (96.2)
	26-35 mm	01 (01.0)
	16-25 mm	03 (02.8)
BMI***	Underweight	32 (30.8)
	Normal	53 (51.0)
	Overweight	15 (14.4)
	Obese	04 (03.8)
*ECOG: Eastern Cooperative Oncology Group, **CNS: Central nervous system, ***BMI: Body mass Index		

Anthropometric and blood parameters

The height of the patients ranged from 134 to 183 cm (mean 161.5). The mean mid-upper arm circumference (MUAC), mid-arm muscle circumference (MAMC), and triceps skinfold thickness (TSF) were 24.7, 21.5 cm, and 10.2 mm, respectively, but below average in both genders in all the patients (Table 3).

**Table 3:** Anthropometric and blood parameters of the study patients

Variables	Range	Mean ± SD (Standard Deviation)
Anthropometric parameters		
Height (in cm)	134-183	161.5±10.4
Weight (in kg)	33.6-86	53.4±10.6
Body mass index (kg/m^2^)	11.5-40.9	20.7 ± 4.7
Mid-upper arm circumference (MUAC) (cm)	18-33	24.7± 3.0
Mid-arm muscle circumference (MAMC) (cm)	15.4-29.5	21.5± 2.8
Triceps skinfold thickness (mm)	04-20	10.2± 2.4
Blood parameters		
Hemoglobin (gm/dl)	8.6-16.7	12.3± 1.5
Red cell distribution width (RDW)	11.2-18.4	13.8±1.6
Total leucocyte count (TLC) (mm^3^)	1010-19700	7075.8 ± 3729.4
Absolute neutrophil count (ANC)	650.5-13708	4655.8 ± 2787.9
Absolute lymphocyte count (ALC)	64.3-4052	1536.2 ± 999.0
Neutrophil to lymphocyte ratio (NLR)	0.05-87.4	4.24 ± 8.42
Serum albumin (gm/dl)	1.2-4.8	3.19 ± 0.89
Total protein (gm/dl)	3.2-9.2	6.7 ± 1.2
Blood sugar (random) mg/dl	65-339	102 ± 41.0

Association of BMI with clinical variables

Twelve patients with head and neck cancer had low BMI, followed by eight with gastrointestinal cancer, whereas six patients with breast cancer were obese. Twenty-two and four patients of stage III and stage IV respectively were underweight. The association between stages of the disease, dental status, type of diet, and BMI was p=0.701, 0.216, and 0.422, respectively, and not statistically significant (Table 4).

**Table 4:** Association of BMI with clinical variables of study patients

Variables	Options	BMI Category				F-value	p-value
		Underweight	Normal	Overweight	Obese		
Gender	Male	20	33	7	1	10.246	0.086^NS^
	Female	12	20	8	3		
Cancer site	Breast	04	13	06	02	5.546	0.814^NS^
	Gastrointestinal	08	05	02	00		
	Genitourinary	01	03	00	02		
	Gynaecological	01	03	02	00		
	Head and Neck	12	24	05	00		
	Lung	05	00	00	00		
	Other	00	04	00	00		
	Total	32	53	15	04		
Stage	I	00	01	00	00	11.172	0.701^NS^
	II	06	10	01	01		
	III	22	28	09	03		
	IV	04	13	05	00		
	Unknown	00	01	00	00		
	Total	32	53	15	04		
Dental status	Edentulous	02	05	00	00	7.558	0.216^ NS^
	Intact	22	36	15	04		
	Missing	08	12	00	00		
	Total	32	53	15	04		
Diet	All	00	01	00	00	12.389	0.422^ NS^
	Liquid	01	03	03	00		
	Semisolid	10	07	02	00		
	Solid	20	40	10	04		
	Tube Feed	01	02	00	00		
	Total	32	53	15	04		
NS- Non-significant at 0.05 level							

Association of BMI with anthropometric and blood parameters of patients

The anthropometric parameters MUAC, MAMC, and TSF were associated with changes in BMI and had statistically significant p values of 0.0001, 0.0001, and 0.033, respectively, but not with hemoglobin, RDW, and NLR (Table 5).

**Table 5:** Association of BMI with anthropometric and blood parameters of patients

Variables	BMI Options	N	Mean	SD	F-value	p-value
Mid-upper arm circumference	Underweight	32	22.07	2.13	22.873	0.0001*
	Normal	53	25.61	2.17		
	Overweight	15	26.28	3.39		
	Obese	4	29.13	1.65		
Mid-arm muscle circumference	Underweight	32	19.14	2.06	19.815	0.0001*
	Normal	53	22.35	2.08		
	Overweight	15	22.89	3.19		
	Obese	4	25.24	1.51		
Triceps skinfold thickness (mm)	Underweight	32	9.33	3.20	3.023	0.033*
	Normal	53	10.38	2.02		
	Overweight	15	10.81	1.39		
	Obese	4	12.38	0.48		
Hemoglobin	Underweight	32	12.41	1.90	0.366	0.778^ NS^
	Normal	53	12.34	1.26		
	Overweight	15	12.45	1.28		
	Obese	4	11.60	2.00		
Red cell distribution width (RDW)	Underweight	32	13.86	1.74	0.703	0.553^ NS^
	Normal	53	13.79	1.57		
	Overweight	15	13.53	1.45		
	Obese	4	14.83	1.35		
Neutrophil lymphocyte ratio (NLR)	Underweight	32	5.95	14.95	0.633 -	0.596^ NS^
	Normal	53	3.50	2.00		
	Overweight	15	3.43	1.25		
	Obese	4	3.25	1.34		
Serum albumin	Underweight	32	2.96	0.97	2.670	0.05*
	Normal	53	3.32	0.80		
	Overweight	15	2.99	0.93		
	Obese	4	4.05	0.54		
NS- Non-significant at 0.05 level; *Significant at 0.05 level						

**Figure 1:**
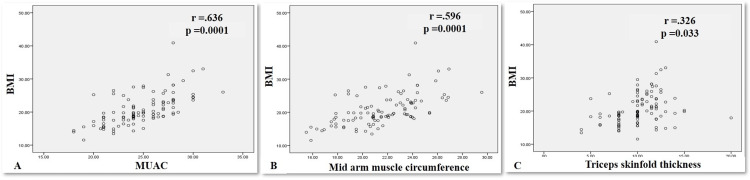
Correlation graphs of BMI with anthropometric parameters (A) MUAC (r=.636; p=0.0001), (B) MAMC (r=.596; p= 0.0001), and (C) TSF (r=.326; p=.033) MUAC: Mid-upper arm circumference; MAMC: mid-arm circumference; TSF: triceps skinfold thickness

The anthropometric parameters MUAC, MAMC, and TSF showed positive correlation with BMI (Figure 1).

## Discussion

Cancer patients are likely to develop nutritional deficiency owing to disease burden and the effect of treatment [6]. The incidence of malnutrition in patients with cancer varies from 40 to 80%, and its causes are multifactorial [7]. It depends on the type of disease, location, stage, treatment received, and method used for nutritional assessment. Also, dietary changes, cancer cachexia, and symptoms having an impact on nutrition are contributory factors [8]. Hence baseline assessment of the nutritional status of cancer patients is very vital. Anthropometric measurements such as weight, MAMC, TSF, and laboratory parameters (such as serum albumin) are frequently used techniques to assess the nutritional status of cancer patients [9]. Hence in the present study, baseline nutritional status of cancer patients was assessed using various anthropometric measurements.

Most common solid tumor sites were included in the present study; however, some, such as sarcoma and melanoma, were not represented. In the present study, we included all stages and sites of cancer disease cases, and overall 30.8% of patients suffered from malnutrition, whereas the study by Muhamed et al. reported around 48.1%, whereas Cuong and Argefa et al. reported 34.1% and 32 % of cancer patients suffered from malnutrition; hence, our study findings were similar to these studies' findings [10-12].

Muhamed et al. reported that the main reasons for malnutrition were low socioeconomic status, different nutritional methods for assessment, lack of adequate healthcare facilities, and dietician support. In our present study, 34.6% of patients with advanced stages (III and IV) presented with poor nourishment and were underweight in the present study. A study by Nourissat et al. reported a strong correlation between weight loss and quality of life in cancer patients [13].

In this study, we found a correlation between the BMI of patients with anthropometric parameters MUAC, MAMC, and TSF. In this study, we also identified a significant correlation of BMI with serum albumin, a widely used laboratory parameter for indices for malnutrition, because of its long half-life [14].

Jeong et al. studied the correlation of blood indices with BMI in children and adolescents. They identified that higher BMI was associated with higher levels of white blood cells (WBCs), red blood cells (RBCs), hemoglobin, hematocrit, and platelet count [15]. The reason for raised WBCs is the production of IL-6 by adipose tissue, which has a role in bone marrow granulopoiesis, and white cell differentiation [16]. However, the reasons for increased RBC indices with obesity are not well understood. Hemoglobin and serum albumin levels have been studied as markers of malnutrition in cancer. The association of blood parameters with BMI in cancer patients has been less studied. In the present study, blood parameters did not correlate with BMI except serum albumin.

The present study had limitations of a small sample size and all solid malignancies were not included. Also, anthropometric measurements (triceps skin fold, midarm muscle circumference) for the assessment of fat deposits and lean body mass are rarely used in a routine clinical setting owing to great variations among individuals and interobserver measurement variability.

## Conclusions

Nutritional assessment is vital in recognizing patients at risk of treatment-associated complications and poor responders to treatment. In this study, BMI correlated with anthropometric parameters MUAC, MAMC, and TSF. Baseline dietary and anthropometric assessments of patients will help to focus on the nutritional build-up of patients before commencement of treatment. 
